# The impact of early adversity and education on genetic and brain morphological predictors of cognitive ability

**DOI:** 10.1111/gbb.12850

**Published:** 2023-07-04

**Authors:** Emma Corley, Laura Fahey, Joan Fitzgerald, Laurena Holleran, Esther Walton, Derek W. Morris, Gary Donohoe

**Affiliations:** ^1^ School of Psychology University of Galway Galway Ireland; ^2^ Centre for Neuroimaging, Cognition & Genomics University of Galway Galway Ireland; ^3^ Discipline of Biochemistry University of Galway Galway Ireland; ^4^ Addiction and Mental Health Group, Department of Psychology University of Bath Bath UK

**Keywords:** childhood adversity, childhood trauma, cognition, education, genome‐wide association, MRI brain volume

## Abstract

Cognitive ability is a strong predictor of occupational achievement, quality of life and physical health. While variation in cognition is strongly heritable and has been robustly associated with early environment and brain morphology, little is known about how these factors combine and interact to explain this variation in cognition. To address this, we modelled the relationship between common genetic variation, grey matter volume, early life adversity and education and cognitive ability in a UK Biobank sample of *N* = 5237 individuals using structural equation modelling. We tested the hypotheses that total grey matter volume would mediate the association between genetic variation and cognitive ability, and that early life adversity and educational attainment would moderate this relationship. Common genetic variation, grey matter volume and early life adversity were each significant predictors in the model, explaining ~15% of variation in cognitive ability. Contrary to our hypothesis, grey matter volume did not mediate the relation between genetic variation and cognition performance. Neither did early life adversity or educational attainment moderate this relation, although educational attainment was observed to moderate the relationship between grey matter volume and cognitive performance. We interpret these findings in terms of the modest explanatory value of currently estimated polygenic scores accounting for variation in cognitive performance (~5%), making potential mediating and moderating variables difficult to confirm.

## Graphical abstract

Genetic variation, early life adversity and total grey matter volume independently predicted cognitive ability. Contrary to our hypothesis, grey matter volume did not mediate the relation between genetic variation and cognition performance. Neither did early life adversity or educational attainment moderate this relation, although educational attainment was observed to moderate the relationship between grey matter volume and cognitive performance.

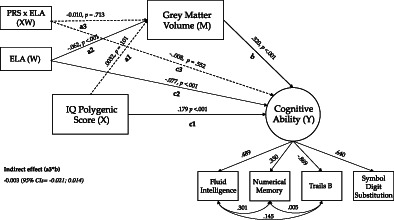

## 1 INTRODUCTION

Cognitive ability is a strong predictor of mental and physical health, as well as mortality.^1^ In the last decade, knowledge about how biological and environmental factors influence cognition has grown rapidly. This includes knowledge about the contribution of genetic variation and environmental factors, as well as the impact of both on brain morphological differences associated with cognition. In particular, the recent availability of large‐scale data, including the UK Biobank, has propelled exciting developments in the area. Despite the significant advances in our understanding of the genetics of cognition, brain structure and environment, we remain at an early stage of modelling how these factors combine and interact. Addressing this gap in knowledge is critical because maintaining cognitive ability in the general population is important for reducing disability across the lifespan. In addition, a substantial body of literature suggests that variability in cognitive functioning can be explained by modifiable risk factors, including that of early life stress and educational attainment.^2^

At a genetic level, twin and family studies have confirmed the heritability of cognitive ability, accounting for about 50% of the total variance in cognition. The contribution of genetics to explaining variation in cognitive ability increases during childhood and adolescence and remains high throughout adulthood.^3^, ^4^ Results from genome‐wide association studies (GWAS) have showed that cognition is highly polygenic, with hundreds of genetic loci of small effect^4^ and that these loci cluster in genes involved in regulating brain‐specific gene expression.^1^ While these effects are small and difficult to detect, the use of polygenic scores (PGS) has made it possible to model the cumulative effects of individual genetic variants and facilitate detection of gene–environment interactions associated with cognition. In the largest study to date,^5^ an intelligence‐based PGS was found to explain up to 5.2% of the variability in intelligence.

A positive association between cognition and brain size has also been robustly identified by multiple studies,^6^, ^7^, ^8^ with both phenotypes sharing a common genetic origin.^9^, ^10^ Indeed, Jansen and colleagues^9^ identified an overlap of 67 genes between both traits with a genetic correlation of 0.23, and the association between genetic variants and cognition has been observed to be partly mediated by grey matter volume (GMV).^10^ In a recent study by Cox et al.,^7^ examining differences in global and regional measures of grey and white matter volume, the strongest contribution to the variance explained in cognitive ability was found for total GMV.

It is widely hypothesised that genetic variation and brain volume both interact with environmental factors to explain variation in cognitive ability.^11^, ^12^ Early life adversity (ELA), including abuse, neglect, witnessing domestic or other violence and chronic poverty,^13^, ^14^ has received a great deal of attention in the literature. Exposure to ELA during periods of heightened plasticity may alter developmental trajectories via structural neurobiological mechanisms that in turn, increase the risk of cognitive impairments in adulthood.^15^ Indeed, in both clinical and non‐clinical samples, ELA has been found to be associated with variability in brain structure, including reductions in total GMV,^16^, ^17^ and across limbic and prefrontal regions^18^, ^19^, ^20^ and several large prospective and retrospective studies have documented an association between ELA and poorer cognitive outcomes in adulthood.^14^, ^21^, ^22^, ^23^, ^24^ Further, in a recent moderated mediation study by Wang et al.,^25^ GMV was found to mediate the association between an intelligence based PGS and cognitive function; this relationship was in turn moderated via ELA, based on data from the Adolescent Brain Cognitive Development study.

In addition to detrimental environmental factors that may moderate the association between genetic variation and cognition, the potential for mitigating factors to buffer the relationship between adverse early experiences and cognitive ability in adulthood, is also poorly understood. In particular, greater years in education has been observed to have positive effects on both cognitive and general health outcomes in individuals exposed to early adverse experiences.^26^, ^27^, ^28^, ^29^ Consequently, the relationship between educational experience and cognitive ability may be causally bi‐directional.^30^, ^31^ Confirming this, however, is made difficult by other genetic and environment factors that likely confound the relationship, including ELA, making it important to model these factors together as potential moderators in the relationship between genetic variation and cognition. Furthermore, understanding how ELA is associated with neurobiological mechanisms underlying cognitive ability and whether these effects can be targeted, that is, via educational attainment, has the potential to inform interventions for individuals exposed to ELA.

The purpose of the present cross sectional study was to examine the moderating role of ELA on the association between genetic variation, total GMV and cognitive ability using the UK Biobank. For this moderated mediation analysis, we used structural equation modelling and generated a latent factor of cognitive ability that was representative of cognitive domains of reasoning, processing speed, working memory and executive function. We hypothesised that the mediating effects of total GMV on the association between genetic variation and cognitive ability would vary depending on the severity of ELA experienced. Genetic variation was indexed using a PGS of Verbal‐Numeric Reasoning, derived from a GWAS we carried out using a non‐overlapping sample of 89,748 UK Biobank participants. Finally, we tested the hypothesis that greater educational attainment would at least in part, the effects of ELA on the association between total GMV, genetic variability and cognitive ability.

## 2 MATERIALS AND METHODS

### 2.1 Participants

The current study used data from the UK Biobank; a large epidemiological cohort study of middle and older age individuals recruited between 2006 and 2010 in the United Kingdom. The study was approved by the National Health Service (NHS) Research Ethics Service (reference 11/NW/0382) and our access to the data was granted by the UK Biobank Access Committee (Project #23739). The total *N* of UK Biobank participants with cognitive and genetic information available was 115,482. This sample was spilt into an adequately powered discovery GWAS sample of 89,748 and a sample of 16,383 used to inform the PGS calculation. The total complete target sample with all available data included (*N* = 5237; age = 38–72 years; 2711 females, 2562 males). The discovery sample subset (*N* = 89,748) of participants were used to carry‐out the Verbal‐Numerical Reasoning GWAS. Selection of the sample and further details of this breakdown are provided in Figure S1 (Table 1).

**TABLE 1:** Descriptive characteristics of the UK Biobank sample.

	Mean (SD)	Range
Age (Years)	56.44 (7.82)	38–72
Education (No college/college)	1742: 3530	‐
Sex (F:M)	2711: 2562	‐
Verbal‐Numerical Reasoning	6.76 (2.03)	0–13
Symbol‐Digit	20.2 (5.04)	0–41
Numerical Memory	6.90 (1.39)	0–12
Trails Making test part B	64.70 (18.73)	20.56–746.53
Grey matter volume (mm^3^)	616,142 (55476.34)	445,943–616,142
IQ polygenic score (*Z* score)	1.52e‐17 (1)	−4.30–3.97
Early life adversity	1.5 (2.073)	0–16

*Note*: Means, standard deviations (SD) and ranges reported, except for frequencies are given for educational attainment and sex.

### 2.2 Cognitive measures

Cognitive tests were administered online on the same day as the MRI scan. As a measure of cognitive ability, we selected four cognitive tests: Verbal‐Numerical Reasoning, Symbol‐Digit Substitution, Matrix Reasoning and Trail Making to maximally capture important domains of cognitive ability including reasoning, processing speed, working memory and executive function. From this we constructed a latent variable of cognitive ability as previous work has shown improvement when combining these tests into a latent variable.^7^, ^32^, ^33^, ^34^

The Verbal‐Numerical reasoning test involved a series of 13 items assessing verbal and arithmetical deduction (Cronbach *α* reliability = 0.62).^35^ The Symbol‐Digit test, which is similar in format to the Symbol Digit modalities test,^36^ involved matching symbols to single‐digit integers and is a well validated measure of processing speed. The score was based on the number of correct Symbol‐Digit matches made in 60 s. For the Numerical Memory test―a measure of working memory―participants were shown a two‐digit number which they had to recall after a short pause. Numbers increased by one until the participant made an error or until they reached the maximum number of 12 digits. In the trail‐making test part B, participants were presented with the numbers 1–13 and the letters A–L arranged pseudo‐randomly on the screen. They were instructed to alternate between touching the numbers in numeric order and letters in alphabetical order (i.e., 1‐A‐2‐B‐3‐C). As detailed by Salthouse^37^ and Cox et al.,^7^ part B of the Trail‐Making test includes both elements of speed and executive functioning. Full details on the content and administration of each test have been published elsewhere.^38^

### 2.3 Education

As a measure of education, UK Biobank participants were asked which of the following qualifications applied to them (with the option of selecting more than one), (1) ‘college or university degree; (2) A levels or AS levels or equivalent; (3) O levels or GCSE or equivalent; (4) CSEs or equivalent; (5) NVQ or HND or HNC or equivalent; (6) Other professional qualifications, for example, nursing, teaching/none of the above; (7) prefer not to answer’. Following the approach described by Rietveld et al.,^39^ we created a binary variable for education to index whether participants had obtained a college or university‐level degree.

### 2.4 Early life adversity

Childhood adversity items were based on the short version of the Childhood Trauma Questionnaire Short Form (CTQ‐SF).^40^ The CTQ‐SF is a self‐report questionnaire measuring physical abuse, emotional abuse, sexual abuse, physical neglect and emotional neglect. Items that were included in the analysis consisted of the following: ‘When I was growing up…’ ‘…I felt loved’ (Loved as a Child): ‘…people in my family hit me so hard that it left me with bruises or marks’ (Physical Abuse); ‘…I felt that someone in my family hated me’ (Hated as a Child); ‘…someone molested me (sexually)’ (Sexual Abuse) and ‘There was someone to take me to the doctor if I needed it’ (Physical Neglect). All questions were rated on a Likert 5‐point scale: never true, rarely true, sometimes true, often, very often true. Additionally, there was the option: ‘prefer not to answer’, which was recoded as a missing value. Scores ranged from 0 to 20, with higher values representing more frequent adversity events. Given the non‐normality of these scores, ELA was log transformed. Distribution and frequency of these scores are detailed in Figure S2.

### 2.5 MRI Acquisition and analysis

MRI data were collected in a single Siemens Skyra 3 T scanner with a standard 32‐channel hear coil located at UK Biobank's recruitment centre. T1‐weighted MPRAGE data was acquired in the sagittal plane using a three‐dimensional magnetization‐prepared rapid gradient‐echo sequence at a resolution of 1 × 1 × 1 mm, with a 208 × 256 × 256 field of view. Global and regional brain imaging‐derived phenotypes (IDPs) were processed by the UK Biobank team and made available to approved researchers. Full details of the brain imaging protocols, and quality control (QC) measures have been made available.^41^ For our study, we used a global brain IDP of total GMV, which had been extracted using FMRIB's Automated Segmentation Tool (FAST).^42^ Scans of individuals with severe and visual normalisation problems were excluded by the UK Biobank through manual inspection (as noted in Alfaro‐Almagro et al.,^41^).

### 2.6 Genome‐wide association analysis of intelligence

We performed a GWAS of intelligence using the Verbal‐Numerical reasoning test from the UK Biobank (discovery sample *N* = 89,748). We chose to conduct our own GWAS primarily to avoid sample overlap in the PGS analysis. The Verbal‐Numerical Reasoning test rather than all four available tests was chosen to maximise the number of participants. However, in agreement with previous authors (e.g., Hagenaars et al.,^35^) we acknowledge that basing our GWAS on the Verbal‐Numerical Reasoning test, in isolation, is a limitation of the study. Cognitive tests available in the UK Biobank are restricted, in that not all participants had completed the same number of tests. For comparison, data on all four tests were available for only 36,383 participants which would not be sufficiently powered to calculate genome‐wide summary data for a PGS of cognitive ability. Notwithstanding, when we examined the degree of correlation between our GWAS beta values to that of previously published GWAS of intelligence (Sniekers et al.,^43^) we found a strong positive correlation between these values.

Genotype data was collected and imputed by the UK Biobank team. Full details of these along with imputation procedures are available in a publication.^44^ In addition to the QC steps performed by the UK Biobank, we removed SNPs on the basis of SNP missingness >0.02, Hardy–Weinberg equilibrium <1 × 10–6 and imputation quality score <0.9. Further, multi‐allelic SNPs were removed as well as SNPs differing in allele frequencies across the genotyping arrays (UK BiLEVE, UK Biobank axiom arrays). The sample was restricted to those of European ancestry which were identified using principal component analysis and 1000 genomes Project (1KGP) data. The multi‐mean of the top eight PCs for 1KGP samples of European ancestry (identified via CEU code) was calculated and participants with a Mahalanobis distance <6 SD from this multi‐mean were considered to be of European descent. Samples were also removed based on the following: relatedness, discordant sex info, high heterozygosity/missingness, chromosomal aneuploidies or retracted consent. Following QC, a total of 7,829,832 variants and 89,748 individuals (41,895 males and 47,853 females) were included. We used a linear regression model in the discovery sample to test for genetic association with intelligence using PLINK 2 (https://www.cog-genomics.org/plink/2.0/). For the analysis, age, gender, genotyping array, UK Biobank assessment centre, socioeconomic status (as assessed by the Townsend Deprivation Index) and the top 10 principal components of genetic population structure were entered in the linear regression.

### 2.7 PGS calculation

We performed a PGS analysis based on our GWAS of Verbal‐Numerical Reasoning (herein referred to as IQ‐PGS) using PRSice‐2.^45^ To avoid potential bias induced by sample overlap, we generated these scores in an independent sample of 16,383 UK Biobank participants (not included in our GWAS) for whom cognitive, genomic and adversity measures were available. SNPs in high linkage disequilibrium were clumped according to PRSice‐2 guidelines. Following this a total of 604,290 variants were included for analysis. An effect‐size weighted PGS was then computed for each individual based on a threshold of *p* < 0.05 with the total number of variants used to inform the PGS being 37,052. While previous studies have used multiple PGS thresholds, we chose a *p* value of 0.05 as this has been shown to maximally capture polygenic risk across a large number of independent samples.^46^ Additionally, we have previously observed this threshold to be the most informative.^47^

### 2.8 Statistical analysis

Structural equation modelling (SEM) calculations were performed in R (version 4.1.2) using the Lavaan package.^48^ To improve convergence, before conducting the SEM analyses, the neural, genetic and cognitive measures were standardised. Missing data were assumed to be missing at random and analyses were conducted using full information maximum likelihood (FIML) estimation. Model fits were assessed with the comparative fit index (CFI), the Tucker–Lewis index (TLI), the root mean square error of approximation (RMSEA) and the standardised root mean square residual (SRMR). The following conventional cut‐off values were used to determine acceptable model fit: CFI > 0.90, TLI > 0.90, RMSEA < 0.06 and SRMR < 0.06.^49^

**FIGURE 1:**
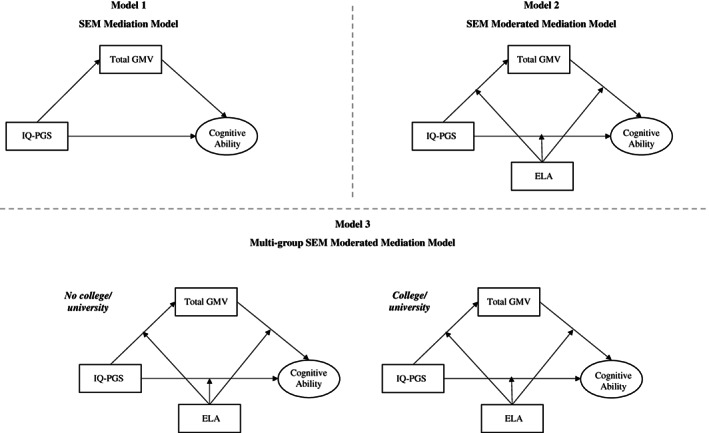
Schematic overview of the SEM models tested. Model one (mediation model) examines the mediation effect of total grey matter volume (GMV) on the relationship between IQ‐polygenic scores (PGS) and cognitive ability. Model 2 (moderated mediation) examines the moderating role of early life adversity (ELA) on IQ‐PGS, total GMV and cognitive ability (direct and indirect pathways). Model 3 (multi‐group SEM model) examines these associations across different educational groups: individuals without (group one) and with a college/university degree or higher (group two).

We first assessed the mediating role of total GMV on the association between IQ‐PGS and cognitive ability. Cognitive ability was defined as a latent construct using the four cognitive tests (see Section 2.2). We further examined whether this association was better captured by the cognitive latent variable or the Verbal‐Numerical Numerical Reasoning test alone. We next evaluated whether ELA would moderate (a) the association between IQ‐PGS and cognitive ability, (b) the association between total GMV and cognitive ability and/or (c) the mediation effect. Finally, we investigated whether the structural model differed across educational groups. See Figure 1 for a schematic overview of the models.

The moderating effect of education (college/university degree vs. no college/university degree) was carried out using a multi‐group SEM, which allowed for the estimation of measurement invariance (factor loadings and path coefficients) across the two groups. Given the sensitivity of the Δ*χ* ^2^ to sample sizes, differences to reject invariance across specifications were based on the following indices and values only: ΔCFI ≤ 0.01 and ΔRMSEA ≤ 0.015 for both factor loadings and intercepts and ΔSRMR ≤ 0.03 for factor loadings and ≤0.01 for intercepts.^50^, ^51^ Throughout models tested, we corrected for age, sex and total intracranial volume (TIV).

## 3 RESULTS

### 3.1 Is the relationship between the IQ‐PGS and cognitive ability mediated by total GMV?

Testing our first hypothesis, which examined the mediating role of total GMV on the association between IQ‐PGS and cognitive ability, we found that the data had two fit indices (TLI and SRMR) outside our pre‐registered criteria (CFI = 0.913; TLI = 0.864; RMSEA = 0.045, SRMR = 0.069). Modification indices (>10) suggested the inclusion of residual correlations between (1) Verbal‐Numerical Reasoning and Numerical Memory, (2) Verbal‐Numerical Reasoning and Trails‐Making Part B and (3) Trails‐Making Part B and Numerical Memory. Following the addition of these residual correlations, the data fitted the model well (CFI = 0.994; TLI = 0.982; RMSEA = 0.016, SRMR = 0.032).

**FIGURE 2:**
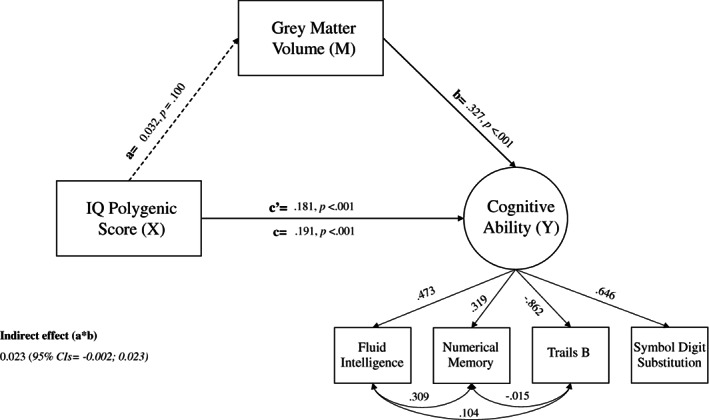
SEM mediation model. The association between the IQ based polygenic score on intelligence as mediated via total grey matter (GM) volume. *X* refers to the predictor variable (IQ‐polygenic scores, PGS), *M* the mediator variable (total grey matter volume, GMV) and *Y* the outcome variable (cognitive ability). Values are standardised beta estimates. Age, sex and total intracranial volume were corrected for. Significant paths are denoted by solid lines and non‐significant paths are dotted lines.

In this model which covaried for the effects of age, sex and TIV, the percentage of variance explained by IQ‐PGS and total GMV on the latent variable of cognitive ability was 14%. By comparison, the percentage of variance explained in the model when the latent variable of cognitive ability was replaced with the VNR test alone was 7%. All associations with cognitive ability were in the expected positive direction, with the largest association being observed for total GMV (*β* = 0.327, SE = 0.006, *p* < 0.001) followed by IQ‐PGS (*β* = 0.181, SE = 0.006, *p* < 0.001). However, the association between the IQ‐PGS and total GMV was nonsignificant (*β* = 0.032, SE = 0.020, *p* = 0.100), and the relationship between IQ‐PGS and cognitive ability was not mediated by total GMV (*indirect effect β* = 0.023, 95% CIs = −0.002; 0.023, SE = 0.006, *p* = 0.096). IQ‐PGS accounted for 3% of the variance explained in cognition ability. Results of these associations are shown in Figure 2.

### 3.2 Does ELA moderate the effect of IQ‐PGS and/or total GMV on cognitive ability?

**FIGURE 3:**
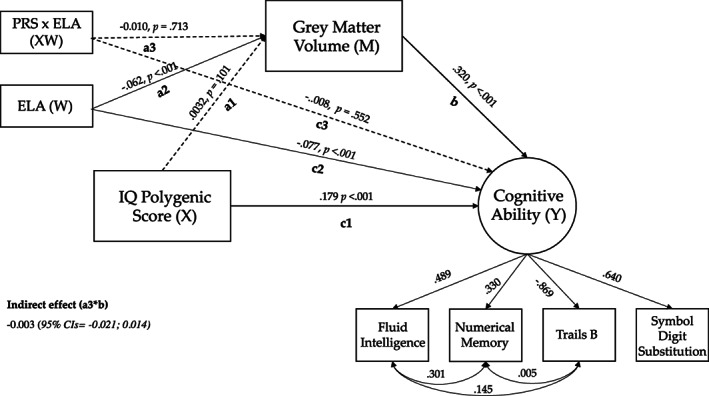
SEM moderated mediation model. The moderating effect of early life adversity on the IQ based polygenic score on cognitive ability as mediated via total grey matter (GM) volume. *X* refers to the predictor variable (IQ‐polygenic scores, PGS), *M* the mediator variable (total grey matter volume, GMV), *W* the moderator variable (early life adversity, ELA) and *Y* the outcome variable (cognitive ability). Values are standardised beta estimates. Age, sex and total intracranial volume were corrected for. Significant paths are denoted by solid lines and non‐significant paths are dotted lines.

Next, we sought to determine whether any differences in structural relationships were influenced by exposure to ELA. Age, sex and TIV was included as covariates of no interest. The results of this model fit the data well (CFI = 0.987; TLI = 0.974; RMSEA = 0.013, SRMR = 0.029), and in total, explained 15% of the variance in cognitive ability. As in the previous model, the direct effects of IQ‐PGS and total GMV on cognitive ability were both significant (Figure 3). In addition, a direct negative association was found between ELA and GMV (*β* = −0.062, SE = 0.010 *p* < 0.001) and between ELA and cognitive ability (*β* = −0.077, SE = 0.006, *p* < 0.001). Contrary to our hypothesis, ELA was not a significant moderator in this moderated mediation model (*indirect effect β* = −0.003, 95% CIs = −0.021; 0.014, SE = 0.009, *p* = 0.714).

### 3.3 Does education moderate the effects of ELA on cognitive ability, either directly or via its effects on IQ‐PGS and/or total GMV?

Our final model examined whether education moderated the effects of adversity on cognitive ability through its association with IQ‐PGS and/or total GMV. Here, the model fit the data well (CFI = 0.967, TLI = 0.949, RMSEA = 0.018, SRMR = 0.034), and the variance explained in cognitive ability was comparable to those with (14.3%) and without (13.5%) a college/university degree. Before testing for group differences, we tested for measurement invariance between the educational groups by (1) constraining the loadings (metric invariance) and (2) the loadings and intercepts (scalar invariance) to equality (tested with analysis of variance, Table 2). In all models, acceptable fit indices were observed and we found that metric and scalar invariance were held across groups. Thus, any observed differences in structural relations were not because of differences or errors in measurement and we proceeded to the multigroup analysis using our baseline model.

**TABLE 2:** Multigroup SEM Model: Testing for measurement invariance across the different educational groups.

	Δ*χ* ^2^ (*p* value)^a^	df	ΔCFI	ΔTLI	ΔRMSEA	ΔSRMR
Configural model			‐	‐	‐	‐
Equal loadings	179.53 (<0.001)	3	0.002	0.001	0	0
Equal loadings and intercepts	977.03 (<0.001)	4	0.009	0.007	0.001	0.006

^a^ We report chi‐square *χ* ^2^ difference for transparency purposes only because of the sensitivity of the Δ*χ* ^2^ to sample size.

As shown in Table 3, IQ‐PGS and total GMV were directly associated with cognitive ability, while the relationship between IQ‐PGS and GMV was nonsignificant in both groups. A significant moderating effect of ELA on the association between GMV and cognitive ability was observed in the group who had been to college/university (*indirect effect β* = 0.015, 95% CIs = −0.022; −0.008, SE = 0.004, *p* < 0.001). However, there was no evidence of a moderated mediation effect of ELA on the association between total GMV, IQ‐PGS and cognitive ability in either group (see Table 3).

**TABLE 3:** Standardised coefficients of moderated mediation model across the educational groups.

	No College/University				College/University			
	*β*	SE	*p*	95% CIs	*β*	SE	*p*	95% CIs
PGS → GM (a1)	0.029	0.024	0.220	−0.018, 0.076	0.010	0.034	0.764	−0.057, 0.077
GM → IQ (b1)	0.304	0.016	<0.001	0.273, 0.336	0.331	0.023	<0.001	0.286, 0.376
PGS × ELA → GM (a2)	−0.013	0.038	0.730	−0.088, 0.061	−0.011	0.043	0.807	−0.095, 0.074
ELA → GM (a3)	−0.049	0.012	<0.001	−0.072, −0.027	−0.016	0.017	0.337	−0.049, 0.017
ELA → IQ (c3)	−0.032	0.006	<0.001	−0.043, −0.020	−0.016	0.009	0.062	−0.034, 0.001
PGS × ELA → IQ (c2)	−0.009	0.017	0.597	−0.043, 0.025	0.008	0.024	0.752	−0.039, 0.054
ELA × GM → IQ (b2)	−0.005	0.006	0.338	−0.016, 0.006	−0.015	0.004	<0.001	−0.022, −0.008
PGS → IQ (c1)	0.179	0.015	<0.001	0.150, 0.207	0.143	0.021	<0.001	0.101, 0.185
PGS → ELA → GM → IQ (a2 × b1)	−0.003	0.014	0.807	−0.031, 0.025	−0.004	0.012	0.730	−0.077, 0.019

Abbreviations: ELA, early life adversity; GM, grey matter volume; IQ, intelligence; PGS, polygenic score.

## 4 DISCUSSION

Based on rich multivariate data from 5273 individuals from the UK Biobank, our study sought to model the complex relationship between genetic variation, GMV, ELA and education on cognitive ability. Consistent with previous studies, we found that genetic variation, as measured by IQ‐PGS and total GMV were each independently predictive of cognitive ability. We did not find evidence that GMV mediated the association between genetic variation and cognitive performance. We further found that ELA was also significantly associated with cognitive performance, but that neither ELA nor years in education moderated the relationship between genetic variation and cognition, either directly or indirectly via GMV.

### 4.1 Brain morphology and the relationship between IQ‐PGS and cognitive ability

Previous studies have found that structural brain metrics are positively associated with cognition,^52^ and that both share a common genetic basis.^9^, ^10^, ^11^ In our study, we focused on total GMV given its consistent, albeit modest, association (~0.15–0.35) with cognition, and because cognitive ability is likely to involve multiple brain areas rather than one specific region.^1^, ^7^ In line with previous studies, IQ‐PGS and total GMV were both predictive of cognitive ability. However, total GMV was not a significant mediator of the relationship between IQ‐PGS and cognitive ability either alone or when moderated by environmental variables.

One interpretation of these findings is that although significant direct associations were observed between cognitive performance and both genetic variation (as measured by the IQ‐PGS) and GMV, the modest amount of variation in cognitive performance explained by genetic variation may have limited our power to detect the mediating role of brain volume. Despite the sample size (>5000 individuals) available for the analysis, a more sensitive measure of brain structure and/or volume may have been required to identify its mediating role. Alternatively, while cognitive variation and GMV were associated, the underlying genetic variation shared between these phenotypes may be insufficient to confirm GMV as a significant mediator of the relationship between an IQ‐PGS and cognitive variation.

### 4.2 Environmental exposure as a moderator of the genetic and brain‐related underpinnings of cognitive ability

In addition to the associations observed between cognitive performance and both polygenic variation and brain volume, exposure to adversity experienced in early life was also significantly associated with cognitive performance. Consistent with previous studies from our group and others, greater exposure to ELA was associated with lower cognitive performance. Contrary to our hypothesis however, ELA was not observed to moderate the relationship between polygenic variance and cognitive performance either directly or indirectly via moderated mediation of brain volume. Educational experience was also not observed to be a significant moderator in the relationship between polygenic variation and cognition, although it was observed to moderate the relationship between GMV and cognitive performance.

Previous studies from our group and others have found evidence of association between ELA, structural variation in GMV and cognitive functioning in both clinical and non‐clinical samples.^17^, ^53^, ^54^ The present study differed from these previous studies by testing whether exposure to ELA represented a potential moderator of genetic effects rather than as an independent variable in its own right. For example, in the Rokita et al., clinical study,^17^ ELA was included as the main predictor of cognitive variation in the model, the effects of which were then observed to be mediated by GMV. For the present study in which genetic variation was the main predictor variable, the relatively small variation in cognitive variation explained by IQ‐PGS coupled with the limited genetic overlap between GMV and cognition may have obscured the moderating effects of childhood adversity. Although this issue might be mitigated by a larger sample size, a more parsimonious conclusion is the explanatory power of the model tested was limited. If true, future studies may benefit from testing alternative models, for example, testing whether polygenic variation might moderate the effects of early environmental exposure to cognitive performance, either directly, or as mediated by a measure of brain volume.

### 4.3 Limitations and strengths

The cross‐sectional design of this study prevents us from drawing any firm conclusions about causality. Indeed, for the models tested here, one could equally speculate about whether individuals with lower cognitive ability and/or socioeconomic status are at greater risk for exposure to ELA, and it will be important for future studies to examine these associations longitudinally. Another potential limitation is that ELA was measured using retrospective self‐reports. However, it is noteworthy that retrospective reports of ELA are shown to correlate moderately well with prospective measures and show comparable effects on negative life outcomes.^55^ Future studies will benefit from the inclusion of a prospective assessment of ELA, as well as considering the developmental timing and frequency of trauma exposure, which may mediate the effects on cognitive functioning.^56^ We did not examine other environmental risk factors (e.g., low birth weight and family income) nor did we include regional specific brain MRI volumes, which may also contribute to variation in cognitive ability. As such, future research should implement a fully data driven approach to include such factors.

Other potential limitations include validity and reliability of the cognitive tests used here, which were brief and administered unsupervised. Notwithstanding, a recent study by Fawns‐Ritchie and Deary^38^ showed that these cognitive tests correlated well with validated standard tests and had a moderate to high test–retest reliability. We further attempted to overcome this limitation by generating a latent variable of cognition comprising of several important domains of cognitive functioning. However, we note that basing our GWAS on the Verbal‐Numerical Reasoning in isolation, may not have been the most informative approach to characterise genetic variation involved in cognitive functioning. Large scale GWAS that comprehensively measure cognition‐ and exclude sample overlap of the UK Biobank‐ are therefore needed to identify the retrospective contribution of these factors. Finally, it is notable that the highest loadings on the cognitive ability factor was for the Symbol‐Digit test and the Trails‐Making test part B; both of which measure executive functioning. Executive functions are worthwhile domain to consider in relation to adversity as they are recognised as an important protective factor for individuals exposed to ELA, promoting better stress and emotion regulation as well cognitive functioning.^57^ Finally, while we controlled for the effects of age, the UK Biobank sample is restricted to middle and older age adults which potentially limits the generalizability of the results.

Our investigation of the relationship between IQ‐PGS, ELA, education and brain morphology on cognition has important strengths, including the relatively large sample size and the inclusion of imaging and genetic data. Further, the SEM approach taken to characterise these associations expands on prior research, which has mostly used simpler regression approaches to analyse the complex relationships between biological and environmental predictors of cognition. The main advantages of SEM are that (1) it considers measurement error among variables, (2) it allows testing complex patterns of relationships and hypotheses simultaneously, (3) it evaluates constructs that cannot be directly measured, (4) allows for residual correlations among variables that may have multicollinearity issues and (5) it can test invariance of effects across different groups. Further, this multivariate technique allowed us to test both multiple direct and indirect effects within an overall model.

### 4.4 Conclusions

This study sought to model the relationship between polygenic variation, environmental exposure, brain volume and cognitive performance in a large non‐clinical sample. While polygenic variation, early adversity and brain volume were each observed to be independently associated with cognitive performance, the hypothesised moderated mediation model was not supported. Given the modest explanatory value of currently estimated PGS, we conclude that future studies modelling the relationship between these variables may benefit from consideration of polygenic variation as itself a moderator of other (e.g., environmental) factors rather than as a predictor variable which is itself moderated.

## AUTHOR CONTRIBUTIONS

*Conceptualization*: Emma Corley, Gary Donohoe, Derek W. Morris, and Laurena Holleran. *Methodology*: Emma Corley, Gary Donohoe, Laura Fahey, and Joan Fitzgerald. *Formal analysis*: Emma Corley and Gary Donohoe. *Investigation*: Emma Corley, Gary Donohoe, Laura Fahey, Joan Fitzgerald, Esther Walton, Laurena Holleran, and Derek W. Morris. *Writing – original draft preparation*: Emma Corley and Gary Donohoe. *Writing – review and editing*: Emma Corley, Gary Donohoe, Laura Fahey, Joan Fitzgerald, Esther Walton, Laurena Holleran, and Derek W. Morris. *Funding acquisition*: Gary Donohoe. *Resources*: Gary Donohoe and Derek W. Morris. *Project administration*: Gary Donohoe and Derek W. Morris. All authors contributed to and approved the final version of the final version of the manuscript.

## CONFLICT OF INTEREST STATEMENT

The authors declare no competing interests.

## Supporting information


**DATA S1.** Supporting Information.

## Data Availability

Data which support the findings of the study can be made available from the corresponding author upon reasonable request.
