# Supplementary material for: The impact of early adversity and education on genetic and brain morphological predictors of cognitive ability
Source: Genes Brain Behav. 2023 Jul 4;22(4):e12850. doi: 10.1111/gbb.12850 (PMC10393420; doi:10.1111/gbb.12850)

**Supplementary Information**

**Supplementary Figure 1 | Analytical N of the UK Biobank Sample**

*Genotype data was quality controlled to remove samples based on non-European ancestry, relatedness, discordant sex information, high heterozygosity and missingness, chromosomal aneuploidies and those who retracted consent. Single nucleotide polymorphisms (SNPs) were removed based on: minor allele frequency (MAF) <0.01, SNP missingness > 0.02, Hardy-Weinberg equilibrium (HWE) ⩽ 1 x 10^-6^ and imputation score <.0.9. Following this, 115,482 participants had cognitive and genetic data and of those, 89,784 samples (discovery samples) were used for our initial GWAS of cognitive ability. Results from this GWAS was then carried forward to generate a polygenic score for the remaining sample of 16,383 UK Biobank participants (target sample). These individuals were selected as they had environmental (early life adversity), education, genetic and at least one measure of cognitive data available. The total N available for participants with data on all measures available was 5,237.*

*
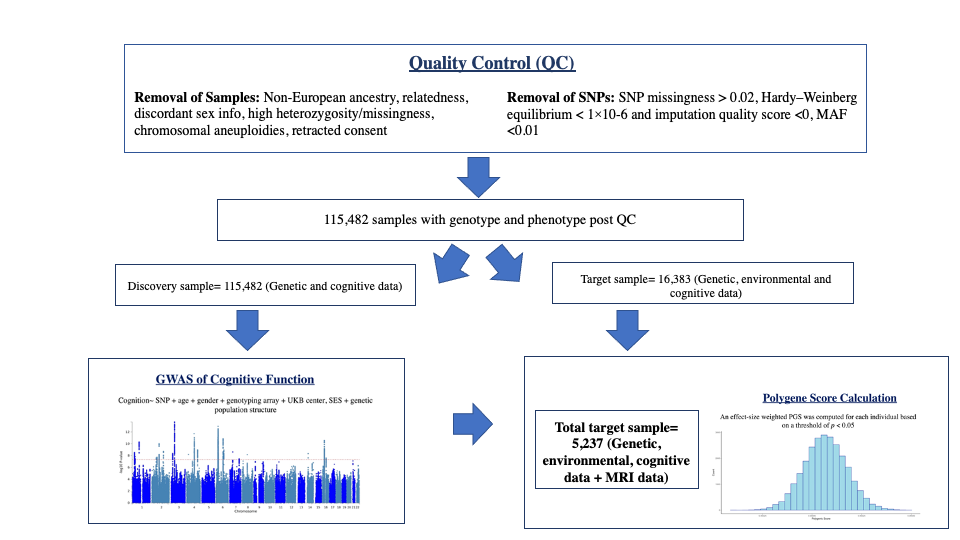
*

**Supplementary Figure 2 | Graphs depicting distribution of early life adversity in the UKB sample**


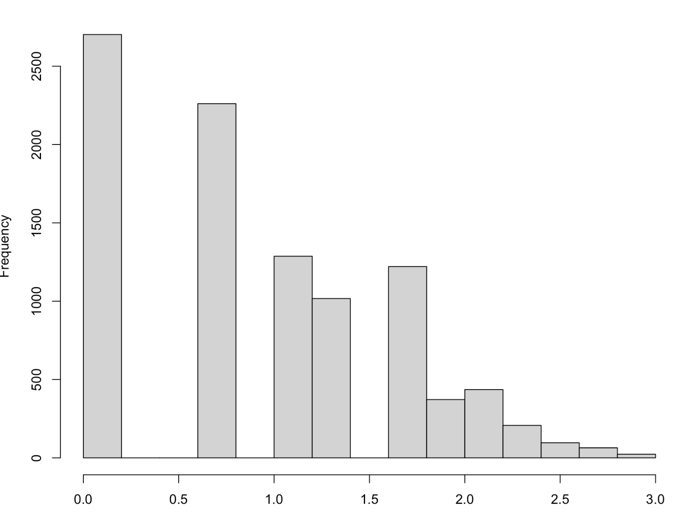

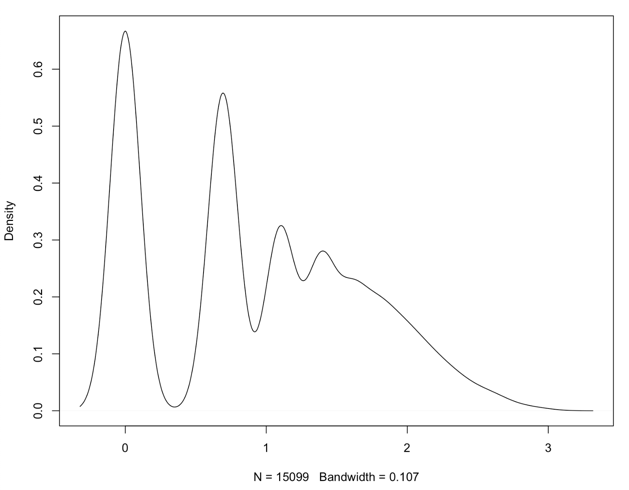

Supplement: Supplementary file 1 — DATA S1. Supporting Information. [file GBB-22-e12850-s001.docx]
